# Lanthanum carbonate hydrate causes artifacts on ultrasound

**DOI:** 10.1007/s00540-015-2034-8

**Published:** 2015-06-07

**Authors:** Kaori K. Kuroiwa, Mana Nakazawa, Masaaki Nishizawa

**Affiliations:** Department of Anesthesia, Nagano Red Cross Hospital, Wakasato, Nagano, 380-8582 Japan

To the Editor:

We experienced a case of high brightness artifacts on abdominal ultrasound sonography (US).

A 66-year-old Japanese male was scheduled for a laparoscopic nephrectomy. He receives haemodialysis from when he was 36 years old 3 times a week for chronic renal failure. Hemodialysis-induced hyperphosphatemia had developed and oral treatment with lanthanum carbonate hydrate (LCH) had been given. General anesthesia was induced, and we attempted a transversus abdominis plane (TAP) block. On the US view, we found a high echoic signal in the intestine ( Supplementary material 1). On abdominal computed tomography (CT), high brightness artifacts were also found in the intestine (Supplementary material 2). Thus, we thought these artifacts were caused by a high-contrast substance in the intestine.

After the operation, a nephrologist told us that the origin of the high-density material in the intestine might be LCH.

LCH is a non-aluminum, non-calcium phosphate binder containing lanthanum, which has been available in Japan since 2009. LCH tablets can be visualized clearly on plain X-ray film and CT [1].

It has been reported that diffuse opacities can be visualized in the intestine on plain X-ray films and on CT in patients taking LCH orally [2]. As well as abdominal US, transesophageal echocardiography is affected by LCH [3]. Most such reports have been made by nephrologists and radiologists. Anesthesiologists perform transesophageal echocardiography and abdominal US, and also need to be familiar with the characteristics of LCH.

## Electronic supplementary material

Supplementary material 1 (JPEG 143 kb)

Supplementary material 2 (JPEG 61 kb)
