# Supplementary figures and images for: Lanthanum carbonate hydrate causes artifacts on ultrasound
Source: J Anesth. 2015 Jun 7;29(6):974. doi: 10.1007/s00540-015-2034-8 (PMC4673092; doi:10.1007/s00540-015-2034-8)

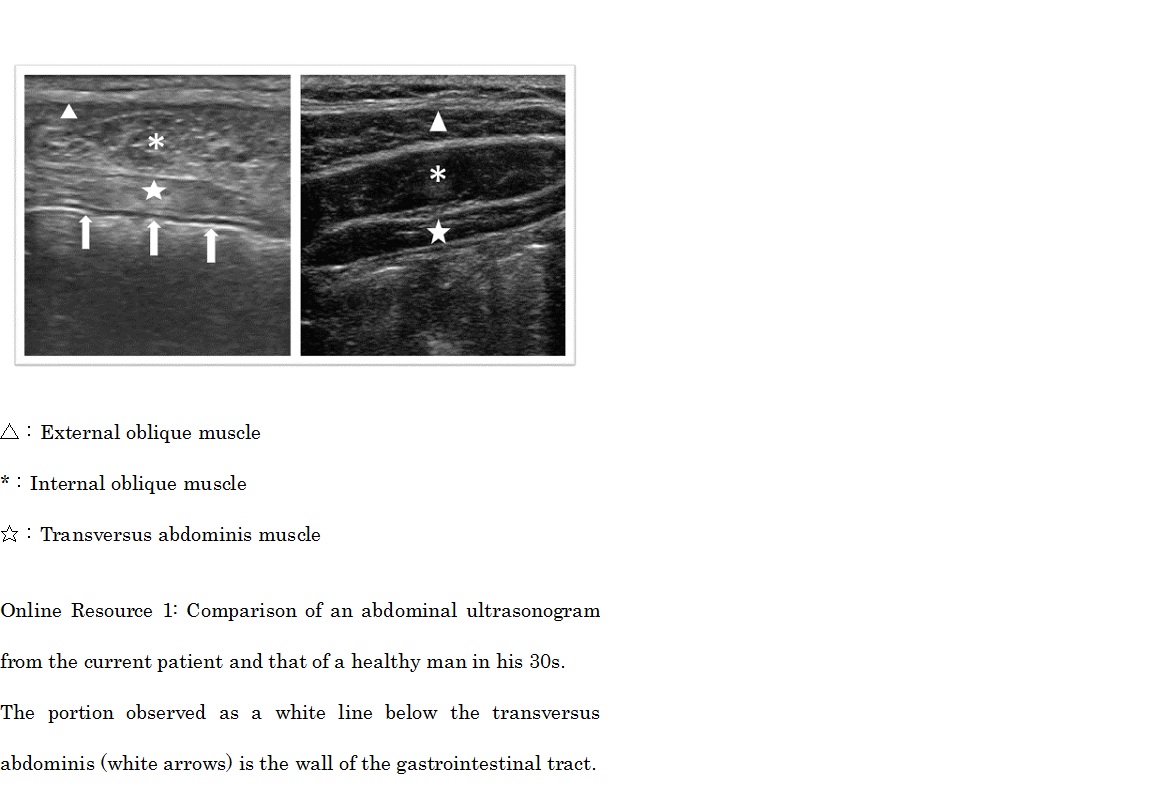

Supplement: Supplementary file 1 — Supplementary material 1 (JPEG 143 kb) [file 540_2015_2034_MOESM1_ESM.jpg]

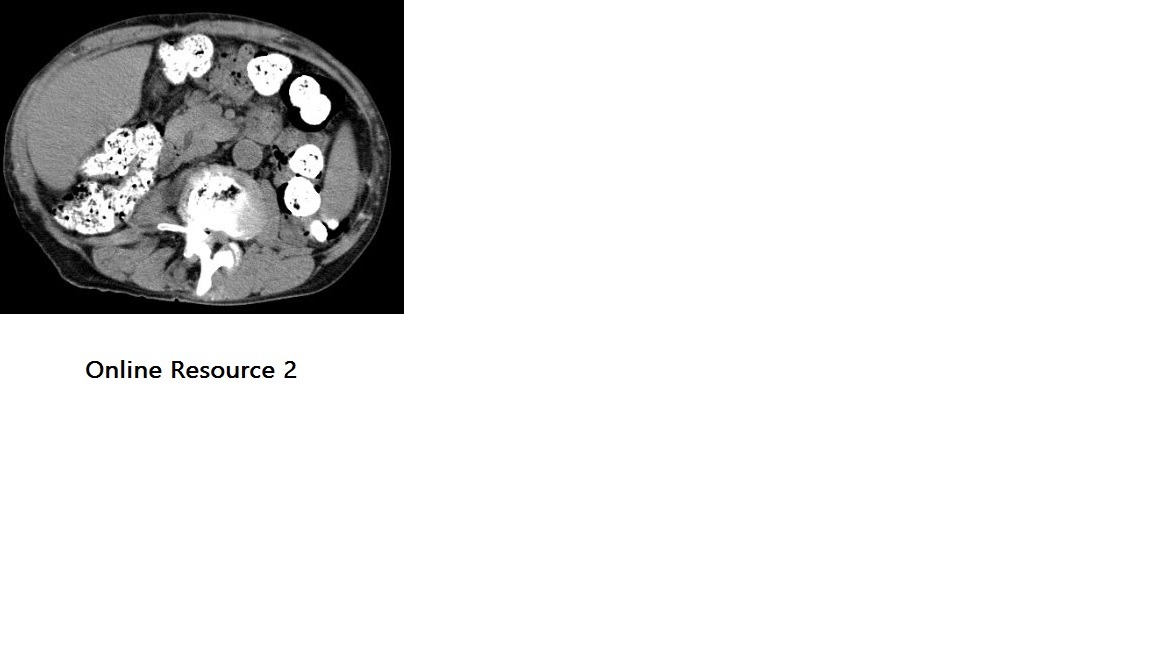

Supplement: Supplementary file 2 — Supplementary material 2 (JPEG 61 kb) [file 540_2015_2034_MOESM2_ESM.jpg]
